# How Does Sex Influence Multimorbidity? Secondary Analysis of a Large Nationally Representative Dataset

**DOI:** 10.3390/ijerph13040391

**Published:** 2016-03-31

**Authors:** Karolina Agur, Gary McLean, Kate Hunt, Bruce Guthrie, Stewart W. Mercer

**Affiliations:** 1 General Practice and Primary Care, Institute of Health and Wellbeing, University of Glasgow, Glasgow, Scotland G12 9LX, UK; karolina.agur@glasgow.ac.uk (K.A.); Gary.McLean@glasgow.ac.uk (G.M.); 2 MRC/CSO Social and Public Health Sciences Unit, University of Glasgow, Glasgow, Scotland G2 3QB, UK; Kate.Hunt@glasgow.ac.uk; 3 Quality, Safety and Informatics Research Group, University of Dundee, Dundee, Scotland DD2 4BF, UK; b.guthrie@dundee.ac.uk

**Keywords:** multimorbidity, sex, primary care

## Abstract

Multimorbidity increases with age and is generally more common in women, but little is known about sex effects on the “typology” of multimorbidity. We have characterized multimorbidity in a large nationally representative primary care dataset in terms of sex in ten year age groups from 25 years to 75 years and over, in a cross-sectional analysis of multimorbidity type (physical-only, mental-only, mixed physical and mental; and commonest conditions) for 1,272,685 adults in Scotland. Our results show that women had more multimorbidity overall in every age group, which was most pronounced in the 45–54 years age group (women 26.5% *vs.* men 19.6%; difference 6.9 (95% CI 6.5 to 7.2). From the age of 45, physical-only multimorbidity was consistently more common in men, and physical-mental multimorbidity more common in women. The biggest difference in physical-mental multimorbidity was found in the 75 years and over group (women 30.9% *vs.* men 21.2%; difference 9.7 (95% CI 9.1 to 10.2). The commonest condition in women was depression until the age of 55 years, thereafter hypertension. In men, drugs misuse had the highest prevalence in those aged 25–34 years, depression for those aged 35–44 years, and hypertension for 45 years and over. Depression, pain, irritable bowel syndrome and thyroid disorders were more common in women than men across all age groups. We conclude that the higher overall prevalence of multimorbidity in women is mainly due to more mixed physical and mental health problems. The marked difference between the sexes over 75 years especially warrants further investigation.

## 1. Introduction

Multimorbidity is usually defined as the co-occurrence of two or more long term conditions within one individual, and is an increasing problem with a reported doubling of prevalence over the last 20 years [1]. It is a global phenomenon and has now become the norm rather than exception in many populations [2,3,4,5]. Multimorbidity is associated with increased mortality [6,7,8], lower quality of life [9,10,11], and greater utilization of healthcare including unplanned admissions [12,13,14] and thus higher healthcare costs [6,15,16,17]. Patients with multimorbidity are less satisfied with care provided [18], perhaps due to the fragmentation of care resulting from the single disease-focus that drives much of current health care [19,20,21].

The prevalence of multimorbidity rises rapidly with increasing age [4,5,6,16,22,23,24], although, the absolute number of individuals with multimorbidity is often higher in those under 65 years [4,25,26]. It is also strongly associated with socioeconomic deprivation and occurs 10–15 years earlier in individuals living in the most deprived compared with the least deprived areas [3,4,5]. The effect of sex on multimorbidity has been less well defined [3]. Most previous studies have shown an increased prevalence of multimorbidity among women [1,6,16,26], though not all studies find this [2,27,28]. Although in almost all countries in the world women have a longer life expectancy than men [29], they are more often affected by a number of non-fatal chronic diseases that decrease quality of life and everyday physical ability [23,30].

Multimorbidity is a broad concept, and can be characterised further in a number of ways, such as physical-only, mental-only, and mixed mental and physical [24], as well as by the commonest combinations of individual conditions. The typology of multimorbidity by sex has not been well documented in large primary care populations. Therefore, this study aimed to examine the relationship between sex and different types of multimorbidity across different age groups, using cross-sectional data from a nationally representative large primary care dataset.

## 2. Methods

We obtained a dataset for 1,751,841 patients of all ages, who were alive on 31 March 2007 and permanently registered with 314 Scottish general practices, from the Primary Care Clinical Informatics Unit at the University of Aberdeen, UK [31]. This accounts for about a third of the Scottish population and is representative of it in terms of age, sex, and socioeconomic deprivation. The NHS National Research Ethics Service had previously approved the use of these anonymised data for research purposes and this analysis did not require independent review [4].

Forty conditions in total were analysed as previously defined [4] using either Quality and Outcomes Framework (QOF) register Read Code sets where available (Read Codes are the clinical coding system used in UK general practice) [32], other Read Code sets used by NHS Scotland for non-QOF conditions, or combinations of Read Codes and prescription data where coding was known to be likely to under-record important conditions. The appendix provides further detail of definitions and the 40 conditions analysed.

Multimorbidity was defined as the presence of two or more disorders in an individual. The choice of conditions was based on recommendations from systematic review for multimorbidity measure [33] as well as diseases in QOF within UK General Practice (GP) contract and important diseases identified by NHS Scotland [32,34]. To specifically examine multimorbidity in terms of physical and mental health disorders, we defined each condition as either a physical or mental health disorder. Of the 40 conditions we classified 32 as physical and 8 as mental disorders (depression, anxiety, drugs misuse, alcohol misuse, schizophrenia/bipolar, dementia, learning disability and anorexia).

In order to characterize the prevalence and type of multimorbidity, we additionally analysed how many people had: (a) 2 or more physical conditions but no mental health conditions (physical-only); (b) 2 or more mental health conditions but no physical conditions (mental-only); or (c) 2 or more conditions including at least 1 physical and 1 mental (mixed physical and mental). We then examined the top 10 most common conditions in those with multimorbidity separately in each age group by sex.

As the current analysis focused on adults with multimorbidity, we excluded adults aged under 25 years in whom multimorbidity is uncommon (prevalence 1.9%) [4]. We divided the remaining patients into 6 age groups (25–34, 35–44, 45–54, 55–64, 65–74 and 75 and over) to reflect different stages in the life course. For each of these age groups, we calculated the number (and percentage) of patients with: no conditions; with one condition; and with two or more conditions. We used graphical display to compare differences by age group in patterns of multimorbidity by sex. We calculated the ten most prevalent conditions by age group and separately for men and women with two or more conditions by dividing the number of patients with each condition by the number of patients in the relevant age groups with two or more conditions. All analysis was conducted using Stata version 13.0 (Stata, College Station, TX, USA).

## 3. Results

The analysis involved 1,272,685 adults aged 25 years and over, of whom 51% were women and 49% were men. Table 1 shows differences in prevalence of morbidity by age group and sex. For both men and women, the number and percentage of people with multimorbidity was higher in each successive ten-year age group within each sex, however, there were more people with multimorbidity in absolute numbers under the age of 65 (201,311 *vs.* 194,996). There were more women with multimorbidity than with a single condition or no condition in all age groups above the age of 55, whereas this was only true in men above the age of 65. The prevalence of multimorbidity (two or more conditions) was higher in women than in men in every age group, although the differences were small over the age of 65. The biggest sex difference was found in the 45–54 age group (women 26.5% *vs.* men 19.6%; difference 6.9 (95% CI 6.5 to 7.2).

Table 2 shows the prevalence of different types of multimorbidity (physical or mental or mixed) across age groups by sex. In general, the differences in prevalence of all types of multimorbidity between men and women were markedly small. However, whilst physical-only morbidity increased with age for both men and women, differences were small up to the age of 64 years but were marginally higher for women. After the age of 65 more men than women had physical-only multimorbidity. The most significant difference was observed in the over 75 age group (women 45.3% *vs.* men 53.5%; difference −8.3 (95% CI −7.7 to −8.7). Gender differences were greatest for mixed physical and mental multimorbidity (as compared with mental-only and physical-only) at all ages but the magnitude of this difference varied with age. Women’s higher prevalence of mixed physical and mental multimorbidity was small in absolute terms at age 25–34 (2.1% (95% CI 1.9 to 2.2), increasing to around 5% between the ages 45 and 74), and highest in those above the age of 75 (women 30.9% *vs.* men 21.2%; difference 9.7 (95% CI 9.1 to 10.2).

**Figure 1:**
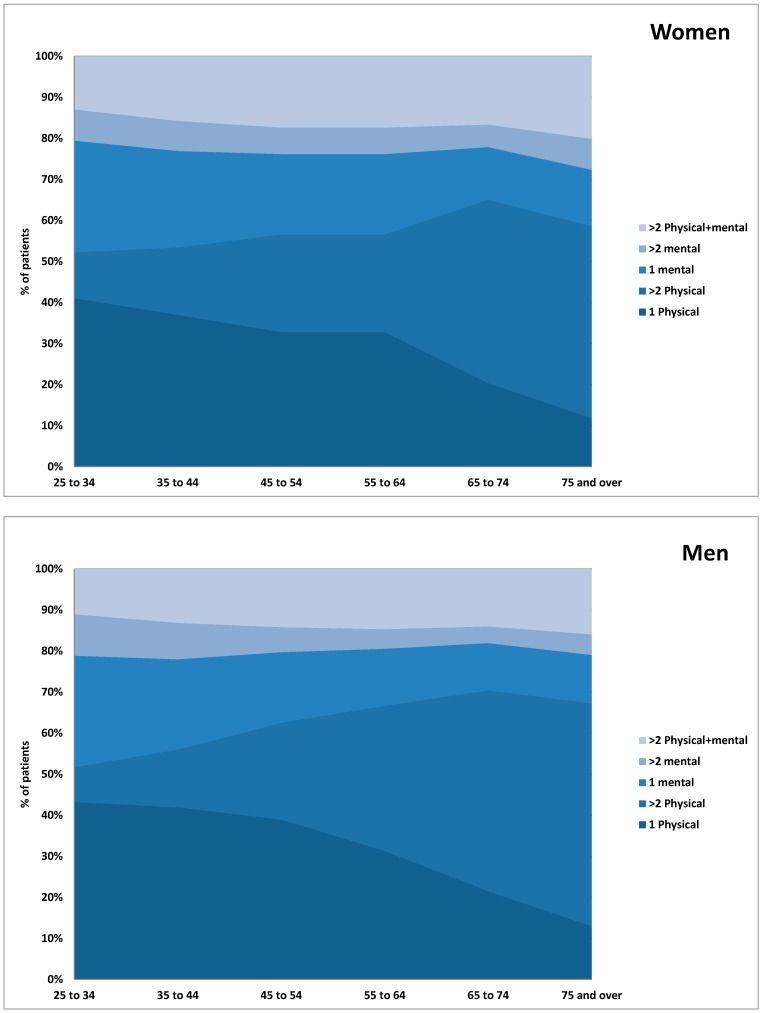
Per cent of people, by type of multimorbidity (physical only, mental only and mixed) and by sex and age.

Figure 1 shows the number of people with the different types of multimorbidity by age group and sex. It illustrates that, overall, women have more multimorbidity across the life course but sex differences are small and diminish even more after 55–64 years. It shows that a large part of this reduction in sex difference is due to a steeper rise in the prevalence of men with physical only multimorbidity compared to women.

Table 3 shows prevalence (%) of the conditions which feature in the top 10 most commonly found in men or women with multimorbidity within each age group. For women with multimorbidity below the age of 55, depression was the condition with the highest prevalence, whereas above the age of 55 hypertension was the most common condition. In contrast for men with multimorbidity, drugs misuse had the highest prevalence for men aged 25–34, depression for men aged 35–44, and hypertension for men aged 45 and over. Women had a consistently higher prevalence of depression, pain, IBS and thyroid disorders across all age groups. For men under 45 with multimorbidity, compared to women in the same age group, prevalence was only notably higher for substance (alcohol and drugs) misuse. Amongst people with multimorbidity, higher prevalence’s of coronary heart disease (CHD) and diabetes were recorded in men than women from the ages of 45 and over.

## 4. Discussion

This study has examined patterns of multimorbidity by age and sex in 1,272,685 adults in a nationally representative primary care sample in Scotland. Women had more overall multimorbidity than men at all ages, although the sex differences were relatively small. From the age of 45, physical-only multimorbidity was consistently more common in men, and physical-mental multimorbidity more common in women. The biggest sex difference in physical-mental multimorbidity was found in the 75 years and over group. The commonest condition in women was depression until the age of 55 years, thereafter hypertension. Depression, pain, irritable bowel syndrome and thyroid disorders were more common in women than men across all age groups.

### 4.1. Relationship with Existing Literature

Several studies have reported that women have more multimorbidity than men [4,26,35], as reflected in a recent systematic review [3]. The level of sex difference in multimorbidity varies between studies. One study reported little difference between the sexes on the risk of being mutlimorbid [36]. This study used 20 chronic conditions and the difference in reported results may be explained by the inclusion in our study of additional conditions which are far more prevalent in women such as migraine and thyroid diseases. Moreover, most previous studies have had either much smaller samples sizes [26,27,36,37] or were restricted to elderly [38,39,40] or hospitalised people [41], whereas the current study examined a nationally representative one-third of the Scottish population.

As far as we are aware, our study is the first to show prevalence rates of different types of multimorbidity and particular conditions by sex and across all ages in a large, nationally representative dataset.

In terms of mental health morbidity, our results show that physical-mental multimorbidity is much more common in women than men. Depression was more prevalent in women than men and was the most frequently occurring condition among women below the age of 55. These findings agree with previously published studies in both multimorbid [4,42] and single disease analyses. [43] Whether this reflects “true” levels of depression in patients with at least one other condition, or a difference in the presentation (by patients) or recognition (by patients and doctors) of depression in people with other long-term conditions cannot be determined from these data. Experimental studies support differential recognition by doctors of potential symptoms in women and men [44,45,46], at least for cardiovascular disease and depression. In addition, below the ages of around 60 years, women are more likely to have contact with their general practitioner [47], and there thus may be differences between men and women of prior recording of depression which may alert GPs to be more alert to depression when they see female patients with physical conditions. Recent survey data have shown that 90% of women with depression discussed it with their doctor [48]. Underestimation of depression in men could perhaps also be explained by so called “masculine” ways of responding to negative affect, less help seeking and lack of consideration for some of such symptoms in commonly used diagnostic tools for depression [49]. This may be important particularly in older populations as previous literature has described that depression is related to negative outcomes within chronic diseases such as, e.g., coronary heart disease [50].

We also found higher rates of alcohol misuse for men with multimorbidity, particularly in the younger age groups, which have also been reported elsewhere [51,52]. This is perhaps not surprising given the consistent finding of higher consumption of alcohol in general population samples of men compared with women in many cultures [53]. It is also consistent with the view that men’s mental distress and depressive illness may disproportionately present as substance abuse [54,55].

Although we did not examine the utilization of healthcare in the current study, increased multimorbidity among female patients, especially those with mental health problems, could explain the previous results in literature in relation to consultation rates and emergency inpatient admissions [12,47]. A recent study using a large dataset in England reported that consultation rate among women was higher than men, which was only partially explained by reproduction related visits [47].

### 4.2. Strengths and Limitations

As far as we are aware, the present study represents the most comprehensive study to date on sex associations with multimorbidity, based as it is on a large nationally representative primary care sample. The study has, however, several limitations. Firstly, it was based on electronic medical records from GP practices and thus relies on the diligence of the practitioners in coding conditions, and their propensity to do this equally diligently by sex for all conditions. We used mixtures of morbidity codes and prescribing to define and measure some conditions, firstly to ensure that coded conditions were still “active” (e.g., asthma code plus recent inhaler prescription) and secondly because some conditions not incentivised in the UK GPs Quality and Outcomes Framework (such as osteoarthritis) are poorly recorded. In addition, the physical conditions we used did not include chronic conditions that are specific to women (in order to compare men and women across conditions). The study is also not able to take account of severity of any of the conditions as these data were not available. Additionally, we did not examine ethnic differences in multimorbidity.

### 4.3. Implications for Policy and Practice

This study shows that multimorbidity differs between men and women in terms of types of conditions as well as prevalence, raising the potential need for a more tailored gendered approach to patients with multiple chronic conditions.

Lack of appropriate guidelines and integrated health systems creates a gap between patients’ healthcare needs and experiences and its provision [55]. As reflected in previous studies, this gap could be decreased by improved integration and a generalist patient-centred approach [56]. Given the apparently large additional multimorbidity burden in women relating to sex-specific conditions, Gender sensitive services development may be important in some settings, including the implementation of sex related health and socio-economic risk factors reduction policies as suggested by WHO [57,58]

Further research is required to understand the differing burdens and impacts of multimorbidity typologies in men and women.

## 5. Conclusions

Our study has shown that, overall, women have more multimorbidity across all age groups, which is driven by a higher prevalence of combined physical and mental multimorbidity in women than men, with the biggest difference in this category being found among the oldest (above the age of 75 year). This warrants further study, including the severity, burden and effects of such sex differences in multimorbidity.

## Figures and Tables

**Table 1:** Number of conditions by age group and sex.

Age Group	No Conditions			One Condition			Two Conditions		
	Women No. (%)	Men No. (%)	Difference (95% CI)	Women No. (%)	Men No. (%)	Difference (95% CI)	Women No. (%)	Men No. (%)	Difference (95% CI)
25–34	78,941 (70.1)	90,806 (77.7)	−7.6 (−7.2 to −7.9)	22,701 (20.2)	18,261 (15.6)	4.6 (4.2 to 4.9)	10,911 (9.7)	7776 (6.7)	3.0 (2.8 to 3.2)
35–44	80,309 (58.9)	99,029 (69.4)	−10.5 (−10.1 to −10.8)	32,966 (24.1)	27,805 (19.5)	4.6 (4.3 to 5.0)	23,025 (16.9)	15,859 (11.1)	5.8 (5.5 to 6.0)
45–54	59,328 (47.3)	72,592 (56.4)	−9.1 (−8.7 to −9.5)	32,751 (26.1)	30,702 (23.9)	2.2 (1.9 to 2.5)	33,230 (26.5)	25,191 (19.6)	6.9 (6.5 to 7.2)
55–64	35,128 (31.9)	40,715 (37.2)	−5.3 (−4.8 to −5.7)	29,120 (26.4)	29,051 (26.6)	−0.2 (0.2 to −0.4)	45,737 (41.6)	39,582 (36.2)	5.4 (4.9 to 5.7)
65–74	14,868 (18.0)	13,794 (18.9)	−0.9 (−0.5 to −1.2)	18,567 (22.5)	16,501 (22.7)	−0.2 (0.2 to −0.5)	49,009 (59.4)	42,541 (58.0)	1.4 (0.5 to 1.5)
75 plus	6989 (8.3)	4864 (9.4)	−1.1 (−0.7 to −1.3)	12,532 (14.9)	8058 (15.5)	−0.6 (−0.1 to −1.0)	64,491 (76.7)	38,955 (75.1)	1.6 (1.2 to 2.1)

Analysis based on 40 chronic conditions; 32 physical and 8 mental, percentages in the conditions columns relate distribution within each separate age group by sex.

**Table 2:** Differences in type of multimorbidity by age group and sex.

Age Group	Physical only Multimorbidity			Mental only Multimorbidity			Mixed Physical and Mental Multimorbidity		
	Women No. (%)	Men No. (%)	Difference (95% CI)	Women No. (%)	Men No. (%)	Difference (95% CI)	Women No. (%)	Men No. (%)	Difference (95% CI)
25–34	3185 (2.8)	1925 (1.6)	1.2 (1.0 to 1.3)	1847 (1.6)	2172 (1.8)	−0.2 (−0.1 to −0.3)	5879 (5.2)	3679 (3.1)	2.1 (1.9 to 2.2)
35–44	7785 (5.7)	5455 (3.8)	1.9 (1.7 to 3.0)	2367 (1.7)	2620 (1.8)	−0.1 (−0.0 to −0.2)	12,873 (9.4)	7784 (5.5)	3.9 (3.7 to 4.1)
45–54	13,999 (11.1)	12,643 (9.8)	1.3 (1.1 to 1.5)	1659 (1.3)	1479 (1.1)	0.2 (0.0 to 0.3)	17,572 (14.0)	11,069 (8.6)	5.4 (5.1 to 5.7)
55–64	25,175 (22.9)	24,596 (22.5)	0.4 (0.0 to 0.7)	891 (0.08)	735 (0.07)	0.01 (0.00 to 0.06)	19,671 (17.9)	14,251 (13.0)	4.9 (4.5 to 5.1)
65–74	31,711 (38.5)	30,825 (42.3)	−3.8 (−3.3 to −4.3)	420 (0.05)	245 (0.03)	0.02 (0.01 to 0.03)	16,878 (20.5)	11,471 (15.7)	4.8 (4.3 to 5.1)
75 plus	38,088 (45.3)	27,824 (53.5)	−8.3 (−7.7 to −8.7)	459 (0.05)	127 (0.02)	0.03 (0.02 to 0.04)	25,944 (30.9)	11,004 (21.2)	9.7 (9.1 to 10.2)

Analysis based on 40 chronic conditions; 32 physical and 8 mental. Percentages are the % in each age group by sex.

**Table 3:** Differences by age and sex in the top 10 most prevalent conditions for multimorbid patients.

Rank Order of Conditions	Age 25–34 (Women) % (95% CI) *n* = 10,911	Age 25–34 (Men) % (95% CI) *n* = 7776	Age 35–44 (Women) % (95% CI) *n* = 23,025	Age 35–44 (Men) % (95% CI) *n* = 15,859	Age 45–54 (Women) % (95% CI) *n* = 33,230	Age 45–54 (Men) % (95% CI) *n* = 25,191
1	Depression 52.9 (51.9–53.8)	Drugs Misuse 38.5 (37.4–39.6)	Depression 53.7 (53.1–54.3)	Depression 36.9 (36.1–37.7)	Depression 46.5 (46.0–47.0)	Hypertension 36.0 (35.4–36.6)
2	Asthma 27.6 (26.8–28.4)	Depression 36.4 (35.3–37.4)	Pain 28.4 (27.8–29.0)	Pain 23.5 (22.8–24.1)	Pain 32.0 (31.5–32.5)	Depression 28.5 (27.9–28.0)
3	Pain 21.4 (20.6–22.1)	Alcohol dependence 22.8 (21.8–23.7)	Asthma 22.6 (22.0–23.1)	Drugs Misuse 22.4 (21.8–23.1)	Hypertension 27.0 (26.5–27.5)	Pain 26.4 (25.9–26.9)
4	IBS 19.9 (19.1–20.6)	Asthma 22.1 (21.1–23.0)	IBS 20.2 (19.7–20.7)	Alcohol dependence 21.8 (21.2–22.4)	Thyroid 19.5 (19.1–19.9)	Alcohol dependence 19.0 (18.5–19.5)
5	Anxiety 19.5 (18.7–20.2)	Anxiety 20.4 (19.5–21.3)	Anxiety 18.2 (17.7–18.7)	Asthma 19.2 (18.6–19.8)	Asthma 18.7 (18.3–19.1)	Dyspepsia 18.9 (18.4–19.4)
6	Drugs Misuse 16.9 (16.1–17.6)	Pain 16.0 (15.1–16.7)	Thyroid 15.7 (15.2–16.1)	Dyspepsia 19.1 (18.4–19.7)	Dyspepsia 18.1 (17.7–18.5)	Diabetes 17.8 (17.3–18.3)
7	Thyroid 11.0 (10.3–11.5)	Dyspepsia 12.4 (11.6–13.1)	Dyspepsia 14.6 (14.1–15.1)	Anxiety 17.2 (16.6–19.8)	IBS 17.8 (17.4–18.2)	Asthma 14.4 (14.0–14.8)
8	Dyspepsia 9.0 (8.5–9.5)	Schizophren/Bi-polar 8.8 (8.1–9.4)	Hypertension 11.6 (11.2–12.0)	Hypertension 16.6 (16.0–17.2)	Anxiety 15.5 (15.0–15.8)	Inflammatory arthritis 12.6 (12.2–13.0)
9	Alcohol dependence 8.6 (8.1–9.1)	Hearing loss 7.9 (7.3–8.5)	Drugs Misuse 9.5 (9.1–9.8)	Diabetes 10.5 (10.0–11.0)	Diabetes 10.7 (10.4–11.0)	Anxiety 11.2 (10.8–11.6)
10	Hearing loss 6.2 (3.2–4.0)	IBS 6.7 (6.1–7.3)	Alcohol dependence 7.4 (7.1–7.7)	IBS 8.0 (7.6–8.4)	Inflammatory arthritis 9.5 (9.2–9.8)	CHD 10.9 (10.5–11.3)
**Rank order of conditions**	**Age 55–64 (Women)** **% (95% CI)** ***n* = 47,727**	**Age 55–64 (Men)** **% (95% CI)** ***n* = 39,852**	**Age 65–74 (Women)** **% (95% CI)** ***n* = 49,009**	**Age 65–74 (Men)** **% (95% CI)** ***n* = 42,541**	**Age 75&over (Women)** **% (95% CI)** ***n* = 64,491**	**Age 75& over (Men)** **% (CI 95%)** ***n* = 38,955**
1	Hypertension 46.6 (46.1–47.0)	Hypertension 50.8 (50.3–51.2)	Hypertension 59.3 (58.8–59.7)	Hypertension 57.1 (56.7–57.6)	Hypertension 64.4 (64.0–64.7)	Hypertension 57.9 (57.3–58.4)
2	Depression 34.7 (34.2–35.1)	Pain 27.8 (27.4–28.2)	Pain 33.5 (33.0–33.9)	CHD 33.2 (32.8–33.7)	CHD 26.6 (26.3–26.9)	CHD 38.8 (38.2–39.2)
3	Pain 34.5 (34.0–34.9)	CHD 22.2 (21.8–22.6)	Depression 23.5 (23.2–23.9)	Pain 25.9 (25.5–26.4)	Pain 26.3 (26.0–26.6)	Diabetes 19.9 (19.5–20.3)
4	Thyroid 21.8 (21.4–22.2)	Diabetes 21.8 (21.3–22.3)	Thyroid 22.4 (22.1–22.8)	Diabetes 24.1 (23.7–24.5)	Thyroid 21.5 (21.2–21.8)	Stroke-TIA 19.2 (18.8–19.5)
5	Dyspepsia 18.5 (18.1–18.8)	Depression 20.3 (19.9–20.7)	CHD 19.9 (19.6 –20.3)	COPD 15.1 (14.8–15.4)	Depression 20.7 (20.4–21.0)	Pain 19.1 (18.7–19.5)
6	Asthma 14.7 (14.3–15.0)	Dyspepsia 15.6 (15.2–16.0)	Diabetes 18.4 (18.1–18.8)	Inflammatory arthritis 14.1 (13.8–14.5)	Chronic Kidney Disease 19.4 (19.1–19.7)	Hearing Loss 18.0 (17.6–18.4)
7	Diabetes 14.2 (13.9–14.5)	Alcohol dependence 14.6 (14.3–14.9)	Dyspepsia 18.0 (17.7–18.4)	Dyspepsia 13.5 (13.1–13.8)	Constipation 18.0 (17.7–18.3)	Chronic Kidney Disease 16.8 (16.5–17.1)
8	Irritable bowel syndrome 13.3 (13.0–13.6)	Inflammatory arthritis 14.3 (14.0–14.6)	COPD 14.2 (13.9–14.5)	Stroke-TIA 12.9 (12.6–13.2)	Dyspepsia 16.4 (16.1–16.7)	COPD 16.6 (16.2–16.9)
9	Anxiety 13.1 (12.8–13.4)	COPD 10.7 (10.4–11.0)	Inflammatory arthritis 13.4 (13.0–13.84)	Depression 12.7 (12.4–13.0)	Anxiety 15.9 (15.6–16.1)	Any Cancer 15.5 (15.1–15.8)
10	Inflammatory poly-arthropathy 12.5 (12.2–12.8)	Asthma 10.2 (9.9–10.5)	Anxiety 13.1 (12.8–13.4)	Hearing Loss 12.2 (11.8–12.5)	Diabetes 15.5 (15.2–15.8)	Constipation 15.2 (14.8–15.5)

Abbreviations: Coronary Heart Disease (CHD); Chronic obstructive pulmonary disease (COPD); Irritable bowel syndrome (IBS).
